# Letter correspondence regarding article “Studying asphyxiation in the lab: the role of experimental evidence in cause-of-death inquiry”

**DOI:** 10.1007/s40656-026-00764-z

**Published:** 2026-09-24

**Authors:** Theodore C. Chan, Gary M. Vilke

**Affiliations:** Department of Emergency Medicine, University of California, San Diego, USA

**Keywords:** Positional, Restraint, Asphyxia

Dear Editors,

We read with interest the article “Studying asphyxiation in the lab: the role of experimental evidence in cause-of-death inquiry” by Fischer and Jukola (Fischer & Jukola, 2026) recently published in the journal *History and Philosophy of the Life Sciences*. The authors unfortunately made a number of erroneous claims regarding the investigative studies conducted by our research group (as well as others) and we appreciate the opportunity to respond and provide a better understanding and history of the research on positional and restraint asphyxia.

Fischer and Jukola attempt to argue that experimental evidence can identify “potential causes” but not “actual causes” of death (or exclude as such) in these cases citing the study by Reay in 1988 as the earliest experimental study suggesting asphyxia as a potential cause based on purported impacts on peripheral oxygenation saturation and heart rate of the prone maximal restraint position (PRMP) in test subjects (Reay et al., 1988). This study had a number of methodological issues including lack of randomization, inadequate measurement of oxygen levels, and statistical errors that led to questions regarding its validity.

The authors then cite our study in 1996 in which we conducted specific respiratory measurements including pulmonary function testing as well as arterial blood oxygen and carbon dioxide (CO_2_) measurements that did not find any impact on oxygenation and CO_2_ levels—a sine qua non for asphyxiation risk (Chan et al., 1997). Our findings clearly refuted the original Reay study upon which rested the experimental evidence of asphyxia as a potential cause. In fact, Reay subsequently declared that the PRMP “should be viewed as not producing significant physiologic respiratory compromise, and it does not produce any serious or life-threatening respiratory effects” (Reay, 1998).

Fischer and Jukola go on to cite our additional experimental work in 2004—a randomized experimental study on PRMP with mild weight force applied—as “a mismatch between methodological design and their claims” (Chan et al., 2004). We vehemently disagree with this characterization. This study again found no detrimental impact in oxygenation or ventilation in subjects, but we were careful (as in all our studies) to cite our limitations including the inability to reproduce all field conditions and the mild amounts of weight applied given that this was our first study investigating the impact of weight. Subsequent studies by our group as well as others with more significant weight force (as much as 225 lbs) have also not shown significant impact on oxygenation or ventilatory physiology (Cary et al., 2000; Michalewicz et al., 2007).

To be fair, Fischer and Jukola state that they do not aim to “assess or evaluate the performed studies”, nor the “methodology or internal validity of the studies” as they “leave such discussions to forensic medical experts”. However, without such critical and expert assessment of these experimental studies (ours and others), one wonders how these authors can then pass comparative judgement, casting such aspersions and accusations of “false claims” and “intended misrepresentations” with their limited understanding of basic physiology and clinical medicine, as well unwillingness and quite frankly, inability to conduct any erudite scientific evaluation.

Finally, it is important to understand the broader context of these studies on multiple levels. First, while Fischer and Jukola might consider “mechanistic evidence” to rank “close to the bottom”, in the history of medicine, much of our understanding of basic human physiology, disease and pathophysiologic processes (such as asphyxia) are largely based on exactly these types of experimental lab studies. Without this evidence, we might not even have the basic understanding or “mechanistic explanation” upon which to consider asphyxia as a “potential cause”.

Second, these studies must also be viewed in the context of other non-laboratory investigations, some of which address the very limitations we acknowledge. Case reports of sudden deaths of restrained individuals (restraint-associated sudden deaths) have been reported in many non-prone positions (sitting, side, supine for example) (Aw-Yong, 2020; Park et al., 2001). Well-performed, prospective large epidemiologic studies from the field have failed to demonstrate an increased risk from prone positioning compared with other restraint positions (Hall et al., 2015; Ross & Hazlett, 2016). As academics and emergency physicians who actively care for patients, we understand this context. It behooves Fischer and Jukola to do the same, lest they themselves be accused of ill-gotten “intentions”, “aspirations” and “false advertising”.
